# Contribution of Early Detection and Adjuvant Treatments to Breast Cancer Mortality Reduction in Catalonia, Spain

**DOI:** 10.1371/journal.pone.0030157

**Published:** 2012-01-17

**Authors:** Ester Vilaprinyo, Teresa Puig, Montserrat Rue

**Affiliations:** 1 Evaluation and Clinical Epidemiology Department, Parc de Salut Mar, Barcelona, Catalonia, Spain; 2 Department of Clinical Epidemiology and Public Health, Hospital de la Santa Creu i Sant Pau IIB-Sant Pau, Barcelona, Spain; 3 Universitat Autònoma de Barcelona (UAB), Catalonia, Spain; 4 Basic Medical Sciences Department, Biomedical Research Institut of Lleida (IRBLLEIDA)-University of Lleida, Lleida, Catalonia, Spain; Univesity of Texas Southwestern Medical Center at Dallas, United States of America

## Abstract

**Background:**

Reductions in breast cancer (BC) mortality in Western countries have been attributed to the use of screening mammography and adjuvant treatments. The goal of this work was to analyze the contributions of both interventions to the decrease in BC mortality between 1975 and 2008 in Catalonia.

**Methodology/Principal Findings:**

A stochastic model was used to quantify the contribution of each intervention. Age standardized BC mortality rates for calendar years 1975–2008 were estimated in four hypothetical scenarios: 1) Only screening, 2) Only adjuvant treatment, 3) Both interventions, and 4) No intervention. For the 30–69 age group, observed Catalan BC mortality rates per 100,000 women-year rose from 29.4 in 1975 to 38.3 in 1993, and afterwards continuously decreased to 23.2 in 2008. If neither of the two interventions had been used, in 2008 the estimated BC mortality would have been 43.5, which, compared to the observed BC mortality rate, indicates a 46.7% reduction. In 2008 the reduction attributable to screening was 20.4%, to adjuvant treatments was 15.8% and to both interventions 34.1%.

**Conclusions/Significance:**

Screening and adjuvant treatments similarly contributed to reducing BC mortality in Catalonia. Mathematical models have been useful to assess the impact of interventions addressed to reduce BC mortality that occurred over nearly the same periods.

## Introduction

Between 1993 and 2007, breast cancer (BC) mortality rates decreased 3% annually in Catalonia [1]. Reduction in BC mortality in Western countries has been attributed to the use of screening mammography and adjuvant treatments [2], and to improved quality of care [3], [4]. Adjuvant treatment is used after primary treatments, such as surgery or radiation, to reduce the risk of relapse. The evaluation of the impact of early detection and adjuvant treatment on BC mortality reduction is challenging due to the fact that both interventions spread almost simultaneously. Mathematical models can overcome this difficulty by estimating BC mortality under different hypothetical scenarios, based on data from the literature and BC registries such as the BC natural history, the stage shift associated with early detection and the benefit attributed to adjuvant treatments in clinical trials [5].

The goal of this work was to estimate the proportion of BC mortality reduction attributable to screening and adjuvant treatments. Previous work from the Cancer Intervention and Surveillance Modeling Network (CISNET) modeled the BC mortality trend in the USA [5]. Based on the work of one of these groups (the Dana-Farber Cancer Institute group) [6], we used a mathematical model to estimate annual BC mortality in the Catalan population under four different scenarios: 1) *Only screening*, 2) *Only adjuvant treatments (tamoxifen and multiagent chemotheraphy)*, 3) *Both interventions*, and 4) *Background* (no intervention).

## Methods

### Mathematical model

BC mortality by age, birth cohort and calendar year was estimated using a stochastic model, originally developed by Lee and Zelen (LZ), that was based on the natural history of the disease [6]. The main assumptions of the model are: 1) BC is a progressive disease with four states: disease-free, pre-clinical (where the disease is present but asymptomatic), clinical (where physical symptoms are present), and death. 2) Women that participate in screening may be diagnosed by a screening exam (annual or biennial) or in the interval between exams, if the disease becomes symptomatic. Women not participating in screening are diagnosed in the clinical state. 3) The benefit of screening is due to a favorable shift in the stage at diagnosis, with a higher proportion of women in early stages relative to usual care (see Section 4 in Appendix S1). In the LZ model, ductal carcinoma in situ (DCIS) cases were not included. The details and the equations for the LZ model can be found in previously published works [6], [7], [8] and in Appendix S1. We previously obtained inputs and modified some equations in the model for the Catalan population [9], [10], [11], [12], [13].

The LZ model takes into account two important biases that emerge when assessing the survival time of screen-detected cancer cases: lead-time and length biases [14]. Lead-time is the length of time between screening detection of a disease and its clinical presentation. Even if early detection had no benefit, the survival of screened individuals would appear longer simply due to the addition of the lead-time. The LZ model is not affected by lead time bias because the screen-detected cases have the origin for which survival is measured at the expected time of clinical diagnosis. The model assumes that the cases diagnosed earlier would have been alive at the time the disease would have been clinically diagnosed. Consequently, there is an implied “guarantee time” for disease-specific survival. Length bias arises because the cancers detected in screening examinations are more likely to have slower growth than symptomatic tumors. The LZ model takes into account the distribution of sojourn time in the pre-clinical state. As a result, exam-diagnosed cases are not a random sample of cases in the pre-clinical state, and tumors with a longer sojourn time in the pre-clinical state are more likely to be detected by screening.

The output of the model was the number of annual BC mortality cases for women aged 30–79 and 30–69 years and born between 1900 and 1975 in the four hypothetical scenarios mentioned above: 1) *Only screening*, 2) *Only adjuvant treatments*, 3) *Both interventions*, and 4) *Background*. BC incidence for women aged less than 30 years is negligible and death certificates for women 80 years or older may have had low accuracy in past decades. Age and cohort specific mortality rates were weighted by the age distribution of the Catalan population at the last year analyzed (2008) to obtain standardized rates for calendar years between 1975 and 2008. Observed annual rates were smoothed using a moving average with window size *k* = 2. The estimations were obtained using the software *Mathematica v7.0*.

### Inputs of the model

#### BC incidence

A previously published age-cohort model that incorporated cohort characteristics like intensity of mammography utilization and fecundity rate was used [9]. This model allowed estimations of BC incidence under the assumption of no screening. Thus, the estimated incidence rates were lower than the observed rates that included overdiagnosed cases. Details and parameters for the incidence estimation can be found in Section 2 in Appendix S1. The estimated incidence was used to derive the probability of transition from the healthy state to the pre-clinical state following the method briefly described in Section 3 in Appendix S1 [15].

#### Mammography sensitivity, sojourn time, and disease stage distributions

Values for *mammography sensitivity* (ranging from 0.35 to 0.8, varying with age and period, see Section 5 in Appendix S1), *sojourn time in the pre-clinical state* (ranging from 2 to 4, varying with age, see Section 3 in Appendix S1), and *stage distributions* were obtained from the literature [6], based on data from the Breast Cancer Surveillance Consortium (BCSC). The distribution of stages at diagnosis was less favorable for the non-screened population than for interval cases or screen-detected cases, and it was less favorable for biennial screening than for annual screening (see Section 4 in Appendix S1).

#### Dissemination of screening mammography

The dissemination of screening mammography by birth cohort had been modeled elsewhere using mixed effects models [11]. The estimates provided the proportion of women that started using periodic mammography at each specific age and the periodicity of exams (annual, biennial and irregular) for mammography users during their lifetimes. Self-declared data was obtained from the three Catalan Health Surveys in the calendar years 1994, 2002 and 2006 [16], [17], [18]. Further details can be found in Section 6 in Appendix S1.

#### Dissemination of adjuvant treatments

Data on the use of adjuvant treatments (multiagent chemotherapy and tamoxifen) during the 1980 s in Catalonia was scarce except for some small studies [19], [20]. Adjuvant treatment was recommended for node positive patients or for high risk node negative patients [21]. The GEICAM group reported the use of adjuvant therapy in Spain in the periods 1990–93 [22] and 1994–97 [23] in two retrospective observational studies that included more than 15,000 patients treated in 43 Spanish hospitals, some of them in Catalonia. These two studies, with some limitations, made it possible to compare the proportion of women with BC that used adjuvant treatments in Spain and the USA, in two cross-sections from the 1990 s. This comparison showed similar levels of treatment use in the two countries during the 1990 s, with higher levels of use in Spain in some periods or stage groups (see Table 1). Based on these results, we assumed that the dissemination of adjuvant treatments during the 1980 s and the 1990 s was similar in both countries. The estimate was taken from Mariotto *et al*. [24], [25].

**Table 1:** Proportion of women with BC who received adjuvant therapies.

	USA*				Spain**			
	1990–93		1994–97		1990–93		1994–97	
	I, II-	II+/IIIa	I, II-	II+/IIIa	I, II, III	II,III	I, II, III	II,III
					(−)	(+)	(−)	(+)
Chemotherapy	0.22	0.36	0.26	0.39	0.26	0.37	0.18	0.23
Hormonotherapy	0.35	0.31	0.36	0.24	0.39	0.28	0.45	0.17
Both	0.08	0.25	0.10	0.30	0.11	0.33	0.25	0.59
None	0.35	0.08	0.28	0.07	0.23	0.02	0.13	0.01

*Obtained from Mariotto *et al*. [24]. In this work chemotherapy was restricted to multiagent chemotherapy and hormonotheraphy was restricted to tamoxifen.

**Obtained from the Alamo I study for years 1990–93 and the Alamo II study for 1994–97 [22], [23].

Positive and negative signs refer to node affectation. Stratification by BC stage groups differs between the two countries.

#### Survival functions

The BC age- and stage-probability density functions (pdfs) for BC survival were assumed to be independent of the mode of diagnosis. The survival distribution of each mode of diagnosis (usual care, screen-detection, interval case) was a mixture of age- and stage-pdfs weighted by the corresponding stage distribution (Section 4 in Appendix S1).

The Catalan survival functions by age and stage for the period 1980–89 were obtained in a previous study [13]. These functions correspond to the pre-screening era in Spain, so they are not affected by lead-time bias.

To introduce the benefit of adjuvant treatments, first the age- and stage-specific Catalan survival functions were adjusted to the mortality reduction reported by clinical trials (Section 4.1 in Appendix S1). Then, the chronological survival pdfs were a mixture of the treatment adjusted pdfs weighted by the proportion of women receiving each adjuvant treatment option, as indicated by the dissemination of adjuvant treatments.

### BC mortality trends under different scenarios

Age-specific BC mortality rates and the standardized mortality rate were estimated for each calendar year in the four hypothetical scenarios, as indicated in the following sections.

#### 1) Background: with no screening and no adjuvant treatments, 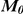


The Catalan 1980–89 survival functions and the stage distributions at BC diagnosis that corresponded to no screening (Section 4 in Appendix S1) were used for women diagnosed at any calendar year of the studied period.

#### 2) Only screening, 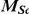


First, BC mortality was estimated assuming that 100% of women from each cohort were in one of the following situations: a) no-screening, b) annual screening (starting at ages 40, 50 or 60) or biennial screening (starting at ages 40, 50 or 60). Second, BC deaths for each birth cohort and calendar year were estimated by taking into account the dissemination of mammography over time. Thus, BC deaths, obtained as indicated in the first step, were weighted by the proportion of women using periodic mammography at the mid-interval of the screening starting at ages 45, 55 and 67.5 years, respectively. According to the observed data, and for all age groups, it was assumed that 65% of the screened women had annual mammograms and 35% had biennial ones [11]. Changes in these proportions changed the final results slightly (data not shown). This process was done using the 1980–89 Catalan survival functions. In this scenario, stage distributions corresponding to screen-detected cases and interval cases, specific for annual and biennial exams, were used (Section 4 in Appendix S1).

#### 3) Only adjuvant treatments, 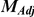


The 1980–89 Catalan survival hazard functions (pre-screening) were multiplied by the hazard ratios corresponding to the benefit of adjuvant treatments (tamoxifen, multiagent chemotherapy or both) and were weighted by adjuvant treatment dissemination in the US, over time. In this way, the effect of the adjuvant treatments modified the pre-screening survival functions towards a more favorable prognosis. In this scenario stage distributions that corresponded to a non-screened population were used (Section 4 in Appendix S1).

#### 4) Both interventions screening and adjuvant treatments, 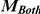


BC deaths under this scenario were estimated by combining the steps described in the *only screening* and the *only adjuvant treatment* scenarios. It is important to notice that when combining screening and adjuvant treatments in the mathematical model there is a synergy between the two interventions, defined as 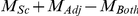. This synergy is negative, meaning that the benefit of screening is larger without adjuvant treatments than it would be with them, and vice versa.

### Mortality reduction

The next step was to assess the benefit of each intervention. For each scenario 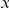, the relative mortality reduction was estimated by comparing the standardized BC mortality rates of the corresponding scenario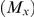 with the Background 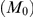. Relative estimates of benefit are less sensitive to misspecification in models and enable to compare different populations [2].

Mortality reductions were estimated at the end of the period (calendar year 2008) using the formula,
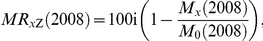
and for the period where BC mortality decreased, since 1990 to 2008, using the formula
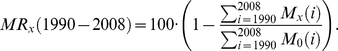



## Results

**Figure 1:**
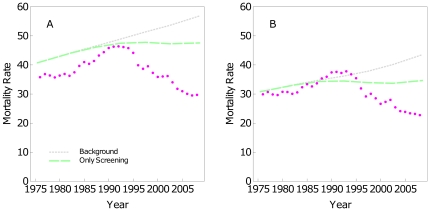
BC mortality rates and screening. Standardized BC mortality rates for the age groups: **A**) 30–79, **B**) 30–69. Observed rates (dots) and estimations under different scenarios *Background* (gray) and *Only screening* (green).

Figure 1A shows BC mortality rates for the age group 30–79 years: observed rates (dots), predictions for the *Background* (gray line) and for the *Only screening scenario* (green line). The model overestimates the observed BC mortality rates. This is not an unexpected result, since the model was not calibrated to reproduce the observed mortality. Adjusting the predicted rates to the observed mortality rate in 1975 would allow to use our model to estimate the impact of screening and adjuvant treatments in the 30–79 age group.

Figure 1B replicates Figure 1A for the age interval 30–69 years. Figure 1B shows an acceptable agreement between observed and predicted mortality rates during the late 1970 s and the 1980 s, when screening and adjuvant treatments were scarcely used in Catalonia. For this reason, we assessed the impact of screening and adjuvant treatments in the 30–69 age interval.

Figure 1B shows that observed BC mortality rates per 100,000 women, 30–69 years old, rose from 29.4 in 1975 to 38.3 in 1993. Afterwards, rates continuously decreased to 23.2 in 2008. The *Background* shows a continuously increasing trend reaching a value of 43.5 in 2008. Compared to the observed rate in 2008 (23.2), the overall percentage reduction in BC mortality at the end of the studied period was estimated as 46.7%.

**Figure 2:**
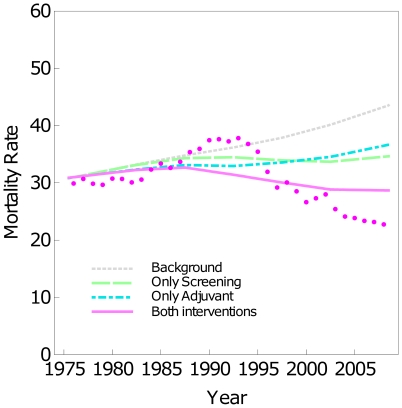
BC mortality rates and different scenarios. Standardized BC mortality rates for the age group 30–69. Observed rates (dots) and estimations under different scenarios *Background* (gray), *Only screening* (green), *Only adjuvant treatments* (cyan), and *Both interventions* (magenta).

Figure 2 adds to Figure 1B the expected trend if *Only adjuvant treatments* had been used (cyan line) and the expected trend if *Both interventions*, screening and adjuvant treatments, had been used (magenta line). Before the 1990 s, mortality under the different scenarios was very similar to the Background. The scenarios *Only screening* and *Only adjuvant treatments* show that screening and adjuvant treatments had similar effects on mortality. The scenario that combines *Both interventions* screening and adjuvant treatments did not fit the observed BC mortality rates. During the late 1980 s and early 1990 s the model-predicted BC mortality underestimated the observed rates. There was agreement during the late 1990 s, but during the 2000 s, the model-predicted BC mortality rates overestimated the observed ones.

Table 2 shows that, at the end of the studied period (year 2008), *Only screening* leads to a 20.4% mortality reduction, *Only adjuvant treatments* to a 15.8% reduction, and *Both interventions* to a 34.1% reduction. This indicates that approximately 3/4 (34.1%/46.7% = 0.73) of the mortality reduction at the end of the studied period can be attributed similarly to screening and adjuvant treatments, and 1/4 remains unexplained. Attributable mortality reductions for the period 1990–2008 were 12.7% for *Only screening*, 12.4% for *Only adjuvant treatments*, and 23.7% for *Both interventions*. Reductions for the 1990–2008 period are lower than the reductions at the end (2008) because they represent an average of the period.

**Table 2:** Percent decline compared to Background for the year 2008 and the period 1975–2008.

BC mortality reduction compared to Background:				
	Only screening	Only adjuvant treatments	Both interventions	Synergy
**2008**	20.4	15.8	34.1	−2.1
**1990–2008**	12.7	12.4	23.7	−1.4

### Sensitivity analysis

A sensitivity analysis was performed to measure the robustness of the results and also to help in understanding the lack of agreement between the observed and estimated rates in the 30–69 age interval during the 1990 s and the 2000 s. Changes in the shape and the level of BC mortality were explored by modifying two of the inputs of the model: survival functions and dissemination of adjuvant treatments.

#### Adding an improvement in the survival functions

**Figure 3:**
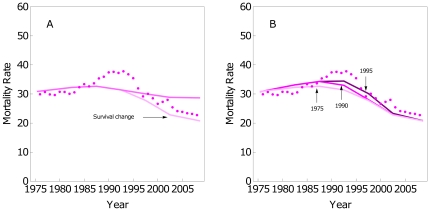
Sensitivity analysis. Standardized BC mortality rates for the age group 30–69. Observed rates (dots) and estimations for *All interventions* scenario. **A**) Changing the BC survival pdfs by the US pdfs for women diagnosed since 1995. **B**) Changing the year of introduction of adjuvant treatments: 1975, 1990, and 1995 (from bottom to top).

The motivation was that other causes than screening and adjuvant treatment seem to play an important role in BC mortality reduction. Survival for Catalan women diagnosed in 1980–89 was worse than for US women diagnosed in 1975–79 (pre-screening era in the US), and the survival functions in Catalonia and the US were similar during the 1990–2001 period. The reasons why the differences in survival between the two countries disappeared are unknown and may be attributed to a plethora of improvements that we refer to as *Other causes*. These may include changes in BC care like the introduction of multidisciplinary teams or changes in population attitudes like higher health awareness. For this reason, the baseline survival functions were changed. An improvement in survival for women diagnosed since 1995 was analyzed by substituting the 1980–89 Catalan survival functions (considered pre-screening in Catalonia) with the 1975–79 US survival functions (considered pre-screening in the USA). Figure 3A shows the BC mortality estimations with and without this improvement in the survival functions. The change in the functions caused that estimations were closer to the observed rates from 1995 to 2008.

To get closer to the observed mortality rates, a second change was added to the improvement in the survival functions.

#### Adding a delay in adjuvant treatment dissemination

The motivation was that the model underestimated the rates around the 1990 s and that using the same dissemination as in the USA could overestimate the use of adjuvant treatments in Catalonia. Figure 3B shows the impact of changing the year when adjuvant treatments began to be used from 1975 to 1990, and to 1995, and assuming that the level of use after the start point was the same as in the US. The results show similar estimated values after the year 2000, and different delays quickly converge to the same levels of BC mortality. The later the introduction of adjuvant treatments, the closer the estimated and the observed rates, improving the shape of the model.

#### Mortality reductions

**Figure 4:**
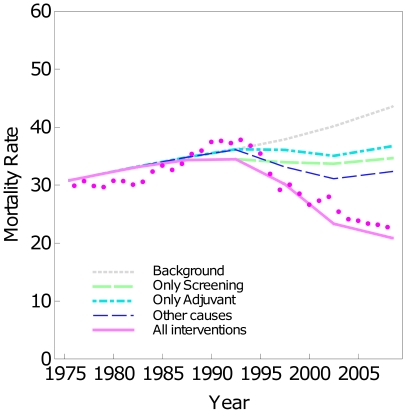
BC mortality rates and different scenarios. Standardized BC mortality rates for the age group 30–69. Observed rates (dots) and estimations under different scenarios *Background* (gray), *Only screening* (green), *Only adjuvant treatments* (cyan), *Other causes* (dark blue), and *All interventions* (magenta).

To assess the robustness of the results we also estimated the BC mortality reductions for the model that best fitted the observed data and which assumes a) the introduction of adjuvant treatments began in 1995 and b) after 1995 the survival pdfs were the US ones. Figure 4 shows the estimated BC mortality rates under the different scenarios for this model. The *Only screening* scenario is the same in Figure 4 as before the sensitivity analysis (Figure 2) because the sensitivity analysis did not change any assumptions about screening. The *Only adjuvant treatment* estimates are higher in Figure 4 because delays in the implementation of treatments cause higher mortalities, but levels in 2008 are similar in Figures 4 and 2.The *Other causes* scenario (blue line in Figure 4) shows that this is the individual scenario with the highest decrease of BC mortality with respect the Background. The *Screening + Adjuvant treatments + Other causes scenario* estimates (magenta line in Figure 4) show agreement with observed rates.

Table 3 shows that the contribution of screening and adjuvant treatments to BC mortality reduction remains stable at the end of the studied period (year 2008). *Only screening* lead to a 20.4% mortality reduction, *Only adjuvant treatments* to a 15.7% reduction, *Other causes* to 25.7%, and *Screening + Adjuvant treatments + Other causes* scenario to a 52.1% reduction. The synergy in the sensitivity analysis was −9.7%.This value is high if compared with the mortality reductions. Synergy increases as more interventions are considered in the model and indicates that once a strategy has a high impact in decreasing the mortality, the potential of other interventions decreases.

**Table 3:** Percent decline compared to Background for the year 2008 and the period 1975–2008.

Sensitivity analysis, BC mortality reduction compared to Background:					
	Only screening	Only adjuvant treatments	Other causes	All interventions	Synergy
**2008**	20.4	15.7	25.7	52.1	−9.7
**1990–2008**	12.7	8.2	15.3	29.4	−6.8

“*All interventions*” includes Screening + Adjuvant treatments + Other causes.

In summary, the mortality estimations obtained with the initial model and after changes in the sensitivity analysis were different, but mortality reductions attributable to screening and adjuvant treatments at the end of the studied period, year 2008, were similar.

## Discussion

### Main findings

Our results suggest that mammography screening and adjuvant treatments have contributed significantly to the reduction in BC mortality in recent decades in Catalonia. For the year 2008, the observed BC mortality rate in the 30–69 age group was around half of the expected rate if none of the two interventions had been introduced (*Background*). The contribution of screening with mammography and adjuvant treatments were similar and together accounted for 3/4 of the overall BC mortality reduction at the end of the studied period. The remaining reduction not explained by the model could be ascribed to other factors, e.g. better BC awareness, advances in surgical procedures or improved health care infrastructure and organization.

The goal of the study was to assess the contributions of screening and adjuvant therapy on BC mortality trends, and this was more closely related to the shape of the trend than to the mortality level. Our initial aim was to assess the impact of screening and adjuvant therapy in women 30–79 years old, but we restricted our analysis to the 30–69 age interval because the predicted mortality rates of our model in the pre-interventions era fitted the data better.

There was also a lack of fit between the observed and expected BC mortality trends in two time periods. Our model underestimated mortality during the late 1980 s and early 1990 s and overestimated mortality during the 2000 s. Possible reasons for not capturing the trend could be 1) the presence of moderate or large errors in some of the inputs or model assumptions, or small errors in most of the inputs. For instance, the fact that the model provides a good approximation for observed data in the pre-screening period for the 30–69 age interval, but not the 30–79 age interval, could be due to the assumption of “guarantee time” between early detection and clinical diagnosis. This assumption may bias the estimated BC mortality rates as women get older, when competing causes of death have a higher impact.

The unexplained portion of the mortality decline can be used to generate hypotheses as to what else was affecting mortality during the studied period. The sensitivity analysis showed that observed BC mortality rates could be better estimated when we changed the model assumptions related to dissemination of adjuvant treatments and survival functions. Several facts can explain this result. First, the model used the US data on adjuvant treatment dissemination. Delays in the dissemination of adjuvant treatment in Catalonia could have affected the measurement of the impact of adjuvant treatments on the observed mortality reduction. Second, the fact that survival in Catalonia during the 1980 s was worse than in the US in 1975–1979, but the differences disappeared during the 1990 s [13], is compatible with improvements in factors other than mammography screening or adjuvant treatments. For example, a stage shift in diagnosis due to greater health awareness or better BC management strategies may have contributed significantly to the observed mortality decline. In Norway the implementation of multidisciplinary teams specializing in BC care was identified as an important element in the reduction of BC mortality [3]. Third, during the 2000 s, there have been improvements in surgery, radiotherapy, and chemotherapy, as well as hormone treatments and biological therapies like trastuzumab [26]. These advances were not taken into account in the initial analysis, and could partially explain the better fit when the survival functions were modified in the sensitivity analysis.

In the majority of Western countries, BC is the malignant tumor with the highest incidence among women (almost 1/3 of all malignant neoplasms). In Catalonia, the decrease in BC mortality rates during the 1990 s was similar to that in other countries in Europe, although the change-points and the mortality levels differed [27]. Since dissemination of screening with mammography and adjuvant treatments overlapped during the 1990 s and the early 2000 s, the separate contribution of each intervention is difficult to assess unless using mathematical models. Our findings are important because they assess the benefit of early detection in the context of continuously increasing survival by using more effective treatments. For instance, the synergism between screening and adjuvant treatment was −1.7. When other causes are also considered in the sensitivity analysis, the synergism increases to −9.7. This shows that the potential impact of reducing BC mortality with screening decreases as long as the contribution of treatments or other causes increases.

#### Comparison with other studies

The model used to estimate BC mortality was developed by the Dana-Farber Cancer Institute researchers Lee and Zelen [6]. These scientists together with six other groups modeled the US BC mortality trend, under the CISNET initiative. The CISNET groups' evaluation of the impact of screening and adjuvant treatments on US BC mortality reduction provided estimates of the percentage decline in the year 2000, ranging from 7.5% to 22.7% for screening with mammography, from 12% to 20.8% for adjuvant treatments, for the 30–79 age interval. Our results for the year 2000 are within the CISNET range, for the 30–79 age group (data not shown). Compared to the USA, one of the differences was the high percent of mortality decline attributable to other causes in Catalonia. In our sensitivity analysis, other causes had a similar contribution than screening and adjuvant treatments. Given that the pre-screening survival functions for Catalonia were worse than in the USA, there was more space for a potential improvement in Catalonia.

Our results differ from the results obtained by Vervoort *et al.* in the Netherlands [28]. They used the computer simulation model MIcrosimulation SCreening ANalysis (MISCAN) to assess the effect of adjuvant therapy and mammography screening on BC mortality. They predicted that the reduction in BC mortality due to adjuvant therapy was 7% in women aged 55–74 years, while the reduction due to screening at 10 years after the screening program was fully implemented would be 28–30%. Of the estimated total BC mortality reduction of 34%, approximately 80% would be explained by screening, whereas 20% could be attributed to adjuvant therapy.

Kalager *et al.* also quantified the effect of screening on BC mortality in Norway [3]. The authors compared BC mortality between periods 1985–1995 and 1996–2005. Among screened women, there was a 28% relative reduction in mortality between the two groups, but screening accounted for only about 1/3 of the total reduction. Although the methods used by the authors and our approximation are completely different, the estimation of the impact of screening on BC mortality reduction is similar and lower than predicted in older clinical trials. The fact that BC treatment has improved considerably in recent years reduces the potential benefits of screening. In an extreme scenario where treatments cured all BC cases, the screening benefit would be negligible.

#### Limitations

This study also has limitations. The main one was the limited information on the dissemination of adjuvant treatments. The two observational studies performed in Spain by the GEICAM group during the 1990 s indicated that the use of adjuvant treatments was similar to or higher than that reported in the USA [29] for the same periods. It could be that doctors participating in the GEICAM studies tended to use adjuvant therapy earlier and/or more frequently than doctors in other Catalan hospitals [22], [23], especially in the late 1980 s and the beginning of the 1990 s when it was not clear if node negative patients would benefit [21]. For this reason, in the sensitivity analysis, it was assumed that a) the use of adjuvant treatments was the same as in the USA after 1990 but was negligible before 1990 and b) the same as in a) but with the time point of 1995. The observed BC mortality rates, higher than those predicted by the model in both situations a) and b), indicated that BC survival in the late 1980 s and early 1990 s was lower in Catalonia than in the US. This result is consistent with a previous analysis by our group [13].

Second, the model relies on data and assumptions that may not be correct. When available, Catalan data from population-based registries or BC screening programs has been used. If the input data was not available at the regional or country level, data from the literature, or that the CISNET had prepared for BC mortality modeling research groups in the US [5], was used. For instance, the stage distribution for screen-detected cases that we have used may be affected by a certain level of overdiagnosis of tumours with limited malignant potential [30], [31]. High levels of overdiagnosis would result in a overestimation of the impact of screening on BC mortality reduction.

Third, confidence intervals for the model outputs were not obtained. Our model is probabilistic because it works with the pdfs of the different inputs related to the natural history or detection of BC. It is also an analytic model that consists of a set of equations describing BC mortality over time. There is uncertainty associated with the model inputs and there is also uncertainty associated with the model structure. It is complex and computationally intensive to obtain the variance of the model estimates. Instead, a sensitivity analysis to explore how changes in the input parameters affect the results was carried out. For example, when the dissemination of the adjuvant treatments was delayed, all the estimations converged to the same levels at the end of studied period. This provides confidence on the robustness of the model.

#### Strengths

To our knowledge, in Europe the number of studies that address the simultaneous quantification of the contribution of mammography screening and adjuvant treatments in BC mortality reduction is scarce. The results obtained are consistent with the estimations of other groups for the USA and Norway, which indicates robustness of the model to departures of the assumptions or to data differences.

It seems that in Catalonia causes other than screening and adjuvant treatments also contributed to BC mortality reduction. The identification of these other causes is challenging and may provide further information for a deeper evaluation of all the interventions that had an impact on the BC mortality reduction.

This work also suggests that some health information registries need to be improved, both at the clinical and population level in Catalonia.

### Conclusion

In conclusion, our study supports the hypothesis that mammography screening, adjuvant treatments and other factors have played an important role in the decline of BC mortality. Approximately 3/4 of mortality reduction can be attributed with similar weight to screening and adjuvant treatments. Probability models have been useful to assess the impact of interventions to reduce BC mortality which occurred over nearly the same periods.

## Supporting Information

Appendix S1 contains details of the mathematical model as well as additional tables and input values.
(PDF)

## References

[1] Perez-Lacasta M, Gregori A, Carles M, Gispert R, Martinez-Alonso M (2010). The evolution of breast cancer mortality and the dissemination of mammography in Catalonia. An analysis by health region.. Revista Española de Salud Pública.

[2] Cronin KA, Feuer EJ, Clarke LD, Plevritis SK (2006). Impact of adjuvant therapy and mammography on U.S. mortality from 1975 to 2000: comparison of mortality results from the cisnet breast cancer base case analysis.. J Natl Cancer Inst Monogr.

[3] Kalager M, Zelen M, Langmark F, Adami HO (2010). Effect of screening mammography on breast-cancer mortality in Norway.. N Engl J Med.

[4] Jorgensen KJ, Zahl PH, Gotzsche PC (2010). Breast cancer mortality in organised mammography screening in Denmark: comparative study.. BMJ.

[5] Feuer EJ (2006). Modeling the impact of adjuvant therapy and screening mammography on U.S. breast cancer mortality between 1975 and 2000: introduction to the problem.. J Natl Cancer Inst Monogr.

[6] Lee S, Zelen M (2006). A stochastic model for predicting the mortality of breast cancer.. J Natl Cancer Inst Monogr.

[7] Lee S, Huang H, Zelen M (2004). Early detection of disease and scheduling of screening examinations.. Stat Methods Med Res.

[8] Lee SJ, Zelen M (2008). Mortality modeling of early detection programs.. Biometrics.

[9] Martinez-Alonso M, Vilaprinyo E, Marcos-Gragera R, Rue M (2010). Breast cancer incidence and overdiagnosis in Catalonia (Spain).. Breast Cancer Res.

[10] Vilaprinyo E, Gispert R, Martinez-Alonso M, Carles M, Pla R (2008). Competing risks to breast cancer mortality in Catalonia.. BMC Cancer.

[11] Rue M, Carles M, Vilaprinyo E, Martinez-Alonso M, Espinas JA (2008). Dissemination of periodic mammography and patterns of use, by birth cohort, in Catalonia (Spain).. BMC Cancer.

[12] Rue M, Vilaprinyo E, Lee S, Martinez-Alonso M, Carles MD (2009). Effectiveness of early detection on breast cancer mortality reduction in Catalonia (Spain).. BMC Cancer.

[13] Vilaprinyo E, Rue M, Marcos-Gragera R, Martinez-Alonso M (2009). Estimation of age- and stage-specific Catalan breast cancer survival functions using US and Catalan survival data.. BMC Cancer.

[14] Zelen M, Feinleib M (1969). On the theory of screening for chronic diseases.. Biometrika.

[15] Lee SJ, Zelen M (1998). Scheduling periodic examinations for the early detection of disease: Applications to breast cancer.. Journal of the American Statistical Association.

[16] Departament de Sanitat i Seguretat Social:Barcelona: Servei Català de la Salut..

[17] Generalitat de Catalunya, Departament de Sanitat i Seguretat Social: La salut i els serveis sanitaris a Catalunya..

[18] Generalitat de Catalunya, Departament de Salut: Enquesta de salut 2006.. http://www.gencat.cat/salut/depsalut/html/ca/plasalut/doc11898.html.

[19] Lluch-Hernandez A, Cervantes-Ruiperez A, Anton-Torres A, Avino-Viguer J, Martinez-Agullo A (1987). Adjuvant chemotherapy with adriamycin, fluorouracil, cyclophosphamide, methotrexate, with or without tamoxifen in operable breast cancer.. Tumori.

[20] Diaz-Rubio E, Martin M, Rosell R, Valerdi JJ, Gonzalez-Larriba JL (1991). The antiemetic efficacy of thiethylperazine and methylprednisolone versus thiethylperazine and placebo in breast cancer patients treated with adjuvant chemotherapy (fluorouracil, doxorubicin and cyclophosphamide). A randomized, double-blind, cross-over trial.. Acta Oncol.

[21] Roman JM, Martin M (1990). [Post-surgical adjuvant treatment of breast cancer].. Rev Clin Esp.

[22] Grupo español de investigación en cáncer de mama (GEICAM) (2002). Proyecto El ÁLAMO I: Encuesta de evolución de pacientes con cáncer de mama en hospitales del grupo GEICAM (1990–1993).. Madrid.

[23] Grupo español de investigación en cáncer de mama (GEICAM) (2004). Proyecto El ÁLAMO II: Encuesta de evolución de pacientes con cáncer de mama en hospitales del grupo GEICAM (1994–1997).. Madrid.

[24] Mariotto A, Feuer EJ, Harlan LC, Wun LM, Johnson KA (2002). Trends in use of adjuvant multi-agent chemotherapy and tamoxifen for breast cancer in the United States: 1975–1999.. J Natl Cancer Inst.

[25] Mariotto AB, Feuer EJ, Harlan LC, Abrams J (2006). Dissemination of adjuvant multiagent chemotherapy and tamoxifen for breast cancer in the United States using estrogen receptor information: 1975–1999.. J Natl Cancer Inst Monogr.

[26] CancerHelp UK (2011). Predicted improvements in breast cancer survival.. http://www.cancerhelp.org.uk/about-cancer/cancer-questions/predicted-improvements-in-breast-cancer-survival.

[27] Gispert R, Cleries R, Puigdefabregas A, Freitas A, Esteban L (2008). [Cancer mortality trends in Catalonia, 1985–2004].. Med Clin (Barc).

[28] Vervoort MM, Draisma G, Fracheboud J, van de Poll-Franse LV, de Koning HJ (2004). Trends in the usage of adjuvant systemic therapy for breast cancer in the Netherlands and its effect on mortality.. Br J Cancer.

[29] Cronin KA, Mariotto AB, Clarke LD, Feuer EJ (2006). Additional common inputs for analyzing impact of adjuvant therapy and mammography on U.S. mortality.. J Natl Cancer Inst Monogr.

[30] Esserman L, Thompson I (2010). Solving the overdiagnosis dilemma.. J Natl Cancer Inst.

[31] Esserman L, Shieh Y, Thompson I (2009). Rethinking screening for breast cancer and prostate cancer.. JAMA.

