# Supplementary material for: Contribution of Early Detection and Adjuvant Treatments to Breast Cancer Mortality Reduction in Catalonia, Spain
Source: PLoS One. 2012 Jan 17;7(1):e30157. doi: 10.1371/journal.pone.0030157 (PMC3260221; doi:10.1371/journal.pone.0030157)
Supplement: Appendix S1 — contains details of the mathematical model as well as additional tables and input values. (PDF) [file pone.0030157.s001.pdf]

APPENDIX S1

1. Natural history of breast cancer and variables of the mathematical model....1

2. Breast cancer incidence model....2

3. Transitions from S_0 to S_p and from S_p to S_c....2

4. Breast cancer survival functions, $g(t)$....3

4.1 Effect of adjuvant treatments in the survival pdf....4

5. Probability of dying of breast cancer, $d_v(y)$....6

6. Dissemination of screening mammography....7

7. References....8

1. NATURAL HISTORY OF BREAST CANCER AND VARIABLES OF THE MATHEMATICAL MODEL

The goal of this Appendix is to provide the values of the inputs of the model and further details of the mathematical equations in order to facilitate the understanding of the main assumptions.

The following Figure A1 shows a simplified scheme of the natural history of the progressive model of breast cancer (BC). The model considers four states: the undetectable or disease free state S_0; the pre-clinical state, S_p, where the disease is asymptomatic but may be detected by screening mammography; the symptomatic disease state, S_c, where BC may detected by usual care; and the state S_d, which indicates death caused by BC. The model defines $w(t)\Delta t$ and $I(t)\Delta t$ as the transition probabilities between S_0 and S_p and between S_p and S_c during an age interval $(t, t+\Delta t)$ where $I(t)$ is the incidence rate function. Figure 1 also shows the ages at which each of the transitions happens, x, τ and y.

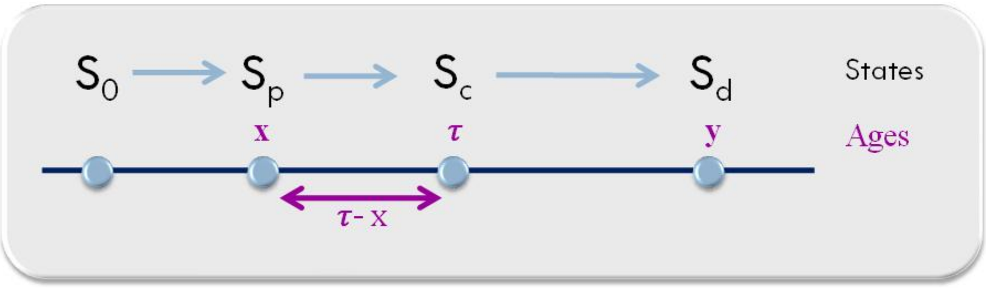

Figure S1. Progressive model of breast cancer.

2. BREAST CANCER INCIDENCE MODEL

Population data on BC incidence, in areas where screening mammography has been used, is affected by early detection. Screening increases incidence rates by age. On the one hand, age at diagnosis decreases as a result of advancing BC diagnosis (lead time). On the other hand, screening may cause overdiagnosis when it detects tumors which would never have been diagnosed during a lifetime without screening. The model developed by Lee and Zelen (LZ model) considers as an input the incidence of the background group, which corresponds to the incidence, by age, in the absence of screening [1]. Then depending on the level of use of mammography the model estimates the number of women that will be diagnosed with BC, taking into account the background incidence, the periodicity of the screening exams, the sensitivity of the screening exams, and the sojourn time in the preclinical state of BC. For this reason, it was necessary to estimate BC incidence in the absence of screening. Reference [2] details the methods we used to estimate the background incidence, which can be summarized as:

a) Estimating an age-cohort Poisson regression model for the observed incidence. The explanatory variables for this model were age at diagnosis and cohort characteristics. Cohort characteristics were completed fertility rate (CFR), percentage of women that used mammography at age 50 (PM50), and year of birth (YB). The model provided the expected BC incidence rate using the following expressions

$$E(I) \ = \ e^{-6.0626 \ - \ 38.8418 \ Age_1 + \ 0.0005 \ Age_2 \ + \ 0.6250 PM50_1 \ + \ 0.0111 YB_1 - \ 0.15 \ CFR}$$

Age_1 = $(age/10)^{-2}$ - 0.0331, Age_2 = $(age/10)^3$ - 166.375, $PM50_1$ = $PM50^{0.5}$ - 0.4342, YB_1 = YB-1937.5.

b) BC incidence in the absence of screening, by cohort of birth, was derived from the Poisson model described in a) when considering that the proportion of women having periodic mammograms at age 50 (PM50) was zero. The incidence rates in absence of screening are lower than the observed ones. Non-progressive disease is not included in the estimated incidence rates.

3. TRANSITIONS FROM S_0 TO S_P AND FROM S_P TO S_C

Since the transition from S_0 to S_p is not observed, the transition probabilities, $w(t)$, can not be estimated directly. Lee and Zelen showed that the, $w(t)$ transition rates can be derived from the S_p $\longrightarrow S_c$ age-specific incidence rates, $I(t)$, and of the sojourn time in S_p, [3]. Let $q(t)$ be the probability density function (pdf) of the sojourn time in S_p, which was assumed to be exponential. For the estimation of $w(t)$, incidence data was grouped by age. Let the jth age interval be defined as $(x_{j-1}, x_j]$ where $x_j - x_{j-1} = 5 = \Delta$. And let $I_j = \int_{x_{j-1}}^{x_j} I(t)\, dt$ be the BC incidence rate for an age group of five years. The transition rate function $w(t)$ was estimated recursively using the formulas

$$w_j = \frac{I_j - Sum_j}{\Delta - m_j(1 - e^{-\lambda_j \Delta})}$$

For the first age interval, $Sum_0 = 0$ and for successive age intervals

$$Sum_j = \sum_{i=1}^{j-1} w_i m_i (e^{-\lambda_i \Delta (j-i)})(e^{-\lambda_i \Delta} + e^{\lambda_i \Delta} - 2)$$

where m_j is the mean sojourn time in S_p and $\lambda_j = 1/m_j$. Values of the mean sojourn time in S_p for different age groups are 2 for ≤ 40 yrs., $-6 + 0.2 \times age$ for (40,50] yrs., and 4 for > 50 yrs. [1]. Young women usually have BC that progress faster than BC in older women.

4. BREAST CANCER SURVIVAL FUNCTIONS, $g(t)$.

Survival is measured from the time of clinical diagnosis in the symptomatic cases and from the estimated time of clinical diagnosis in the screen detected cases. In the LZ model the survival function adjusts for the lead time in the following way. In the LZ model, the exam-detected, interval, and clinical cases all have the origin for which survival is measured at the time of clinical diagnosis. Consequently, there is an implied "guarantee time" for disease-specific survival, that is, the cases diagnosed earlier would have been alive at the time the disease would have been clinically diagnosed. This guarantee time which corresponds to the lead-time is a random variable and is incorporated into the model equations.

In a previous work we derived twenty five BC survival pdfs by age (five age groups) and stage (I,II-, II+,III or IV) at diagnosis for the period prior to the introduction of BC screening and adjuvant treatments 1980-89 [4].

The g is a mixture of five pdfs weighted by the probability of being diagnosed at stage i at age τ ($\theta_{i|\tau}$). One of the assumptions of the model is that the screen-diagnosed cases may have a stage shift towards a more favorable prognosis relative to the distribution of stages with usual care (see [1] for further details). Using the expression

$$g(t|\tau) = \sum_i \theta_{i|\tau} g_i(t|\tau)$$

the BC survival pdfs for the ***Background*** (g^{bg}) and the ***Screening*** scenarios were obtained. For the ***Screening*** scenarios we differentiated between screen-detected (g^{s_a}, g^{s_b}) and interval cases (g^{i_a}, g^{i_b}), where the subindexes a and b refer to annual and biennial periodicities, respectively. The corresponding stage distributions for each of the survival functions can be found in Table S1.

Ages	Stage distributions				
	I	II-	II+	III	IV
Without screening (SEER 1975-79), θ^{bg}					
30-39	0.31	0.23	0.31	0.09	0.06
40-49	0.30	0.23	0.31	0.10	0.06
50-59	0.29	0.22	0.31	0.10	0.08
60-69	0.30	0.22	0.27	0.10	0.11
70-84	0.32	0.27	0.22	0.10	0.10
Annual screening: screen-detected, θ^{s_a}					
< 50	0.62	0.11	0.21	0.04	0.01
50-59	0.67	0.11	0.19	0.03	<0.01
60-69	0.76	0.07	0.14	0.02	<0.01
> 69	0.78	0.09	0.11	0.02	0.01
Annual screening: interval cases, θ^{i_a}					
< 50	0.46	0.19	0.26	0.07	0.02
50-59	0.45	0.17	0.30	0.07	0.01
60-69	0.54	0.15	0.23	0.06	0.01
> 69	0.54	0.23	0.16	0.05	0.01
Biennial screening: screen-detected, θ^{s_b}					
< 50	0.58	0.12	0.24	0.04	0.01
50-59	0.62	0.15	0.17	0.04	0.02
60-69	0.66	0.13	0.18	0.02	0.01
> 69	0.73	0.13	0.11	0.01	0.01
Biennial screening: interval cases, θ^{i_b}					
< 50	0.37	0.22	0.31	0.08	0.02
50-59	0.29	0.26	0.26	0.12	0.06
60-69	0.41	0.22	0.27	0.07	0.03
> 69	0.43	0.29	0.18	0.07	0.03

Table S1. Breast cancer stage distributions, by age. The classification of cancer staging is from the American Joint Committee on Cancer 3rd edition and was obtained from [1].

4.1 EFFECT OF ADJUVANT TREATMENTS IN THE SURVIVAL PDF

As Lee and Zelen proposed [1], the estimation of the impact of ***Adjuvant treatments*** considered six different treatment combinations (tt): only multiagent chemotherapy, only tamoxifen with a duration of 2 or 5 years, both multiagent chemotherapy and tamoxifen, and no adjuvant treatments. The estimated mortality reductions ($\mathrm{MR}_\tau^{\mathrm{tt}}$) for women receiving treatment tt at age τ shown in Table S2 [5] were used to obtain new survival pdfs, each one of the twenty five g_i by solving the following differential equation

$$\frac{\mathrm{d}}{\mathrm{dt}}\,\mathrm{S}_i^{\mathrm{tt}}(\mathrm{t}|\tau) = -\mathrm{HR}_\tau^{\mathrm{tt}}\frac{g_i(\mathrm{t}|\tau)}{S_i(\mathrm{t}|\tau)}\,\mathrm{S}_i^{\mathrm{tt}}(\mathrm{t}|\tau);\;\; \mathrm{S}_i^{\mathrm{tt}}(0) = 1$$

HR_{τ}^{tt} indicates the hazard ratio obtained as $HR_{\tau}^{tt} = 1 - MR_{\tau}^{tt}$, and S_i is the BC survival function $S_i(t|\tau) = 1 - \int_0^t g_i\,(u|\tau)du$. Then the new pdfs were derived as

$$g_i^{tt}(t|\tau) = -\frac{d}{dt} S_i^{tt}(t|\tau)$$

The BC survival pdf for the Adjuvant treatment scenario g^{Adj} is a mixture of the g_i^{tt} weighted by $\phi_i^{tt}(\tau + v|\tau)$ which corresponds to is the probability of receiving treatment tt at calendar year $\tau + v$ for a woman diagnosed at age τ, then

$$g^{Adj}(t|\tau, \tau + v) = \sum_i \sum_{tt} \theta_{i|\tau}^{bg} \phi_i^{tt}(\tau + v|\tau) g_i^{tt}(t|\tau)$$

Note that the stage distribution for adjuvant treatments is the same than for the Background. The ϕ_i^{tt} where obtained from Mariotto et al. [5,6], who modeled the adjuvant treatment dissemination. The benefit of tamoxifen was applied to the percentage of women with estrogen receptor positive BC. Percentages were of 63% (<50 yrs.), 77% (50-69 yrs.) and 85% (≥70 yrs.) (see [1] for more details).

**Multiagent chemotherapy only**		
**Age**		
**< 50**	27	
**50-69**	14	
**60-69**	8	
**Tamoxifen only***		
	**2 years**	**5 years**
	18	28
**Multiagent chemotherapy and tamoxifen***		
	**2 yr. Tam**	**5 yr. Tam**
**< 50**	40	47
**50-69**	29	38
**60-69**	25	34

Table S2. Percentage of reduction in the mortality hazard ratio due to the use of adjuvant treatments. *These values are for estrogen-receptor positive cancers. .

Finally, to obtain the BC survival pdfs for the scenario that incorporates ***Both Adjuvant treatments+Screening*** we followed the same steps as we did for g^{Adj}. The difference was that, instead of using the Background stage distribution, θ^{bg}, we used each of the stage distributions associated with screening (screen detected and interval cases for annual and biennial screening), thus obtaining the survival functions g^{Adj+s_a}, g^{Adj+i_a}, g^{Adj+s_b} and g^{Adj+i_b}.

5. PROBABILITY OF DYING OF BREAST CANCER, $d_v(y)$.

The probability of dying of BC, $d_v(y)$, at age y for birth cohort v for the ***Background*** scenario was estimated as:

$$d_v(y) = \int_0^y \int_0^\tau S_v(x) w_v(x) q(\tau - x|x) g^{bg}(y - \tau|\tau) dx d\tau \qquad (1)$$

where $S_v(t)$ is the probability of surviving to year t for birth cohort v for women from the general population.

Equation (1) was also used to estimate the probability of death for the ***Only adjuvant treatments*** scenario, using the survival pdfs that include the treatment effect. For the Background scenario we used the Background pdf g^{bg} while in the Only adjuvant treatments we used the g^{Adj} pdf that include the benefits of tamoxifen and multiagent chemotherapy and takes into account their dissemination usage conditioned to the period of diagnosis.

The estimation of the probability of dying of BC in the scenarios that include the ***Screening*** benefit is more complex. The full set of equations can be found in [7]. To illustrate it, the next two equations show the probabilities of BC death for women that had only one exam at age z. The probability of BC death for **screen-detected cases** was estimated as:

$$d_v(y|s_a, z) = \beta_{z,z+v} \int_z^y \int_0^z S_v(x) w_v(x) q(\tau - x|x) g^{s_a}(y - \tau|\tau) dx d\tau \qquad (2)$$

The $\beta_{z,z+v}$ is the sensitivity of the mammography exam, which depends on the woman's age and the calendar year at which the mammogram was performed. The values used in the model are shown in Table S3 and were obtained from [1].

The probability of dying of BC for **interval cases** was estimated as:

$$\begin{aligned} d_v(y|i_a, z) = & \left(1 - \beta_{z,z+v}\right) \int_0^y \int_0^z S_v(x) w_v(x) q(\tau - x|x) g^{i_a}(y - \tau|\tau) dx d\tau \\ & + \int_z^y \int_z^\tau S_v(x) w_v(x) q(\tau - x|x) g^{i_a}(y - \tau|\tau) dx d\tau \end{aligned} \qquad (3)$$

Equation (3) has two components: the first refers to women that were in the preclinical state at the time that the exam was performed but the result of the exam was negative (false negative cases); and the second refers to women that entered the preclinical state after the first exam at age z (true interval cases).

When estimating probabilities of BC death for women that had more mammographic exams, the false positive results may take place at any of the previous exams.

The equations that allow estimation of the probability of BC death have the same expression for annual or biennial screening periodicities, with the difference in the survival functions. Each screening periodicity has an associated survival function that takes into account the corresponding distribution of disease stages at diagnosis. Survival functions associated with annual screening are g^{s_a} for screen detected cases and g^{i_a} for interval cases; for biennial periodicity, survival functions are g^{s_b} for screen detected cases and g^{i_b} for interval cases.

The scenario that combines ***Both adjuvant treatments and screening*** is estimated using the same equations as the Only screening scenario. The survival pdfs incorporate the benefits of adjuvant treatments and the stage distribution of screen detected and interval cases for annual and biennial screening: g^{Adj+s_a}, g^{Adj+i_a}, g^{Adj+s_b} and g^{Adj+i_b}.

Age	Period		
	< 1985	1985-1994	≥ 1995
< 40	0.35	0.45	0.55
40-44	0.45	0.55	0.65
45-49	0.50	0.60	0.70
50-69	0.60	0.70	0.75
≥ 70	0.70	0.75	0.80

Table S3. Mammography sensitivity by age and period. Values were obtained from [1].

6. DISSEMINATION OF SCREENING MAMMOGRAPHY

The proportions of women who were having periodic mammographies for early detection in Catalonia by age and birth cohort v was estimated in [8] following a mixed effects model:

$$p_v(age) = \frac{\rho_1}{1 + e^{(\rho_2 - age)/\rho_3}}$$

The ρ_1 is the horizontal asymptote as age increases, ρ_2 indicates the age value at which approximately half of the population is receiving periodic mammograms, and ρ_3 indicates the difference in years between the age at which 3/4 of the population are receiving periodic mammograms and the age ρ_2.

The parameters $\rho_1$and ρ_3 were estimated as fixed effects and the parameter ρ_2 as random effect. Parameter values can be found in [8].

Finally, based on results from a previous publication [8], the probability of receiving annual screening was estimated as $0.637 \times p_v(age)$ and the probability of receiving biennial screening was estimated as $0.363 \times p_v(age)$.

7. REFERENCES

1. Lee S, Zelen M (2006) A stochastic model for predicting the mortality of breast cancer. J Natl Cancer Inst Monogr: 79-86.

2. Martinez-Alonso M, Vilaprinyo E, Marcos-Gragera R, Rue M (2010) Breast cancer incidence and overdiagnosis in Catalonia (Spain). Breast Cancer Res 12: R58.

3. Lee SJ, Zelen M (1998) Scheduling periodic examinations for the early detection of disease: Applications to breast cancer. Journal of the American Statistical Association 93: 1271-1281.

4. Vilaprinyo E, Rue M, Marcos-Gragera R, Martinez-Alonso M (2009) Estimation of age- and stage-specific Catalan breast cancer survival functions using US and Catalan survival data. BMC Cancer 9: 98.

5. Mariotto AB, Feuer EJ, Harlan LC, Abrams J (2006) Dissemination of adjuvant multiagent chemotherapy and tamoxifen for breast cancer in the United States using estrogen receptor information: 1975-1999. J Natl Cancer Inst Monogr: 7-15.

6. Mariotto A, Feuer EJ, Harlan LC, Wun LM, Johnson KA, et al. (2002) Trends in use of adjuvant multi-agent chemotherapy and tamoxifen for breast cancer in the United States: 1975-1999. J Natl Cancer Inst 94: 1626-1634.

7. Carles M, Vilaprinyo E, Cots F, Gregori A, Pla R, et al. Cost-effectiveness of early detection of breast cancer in Catalonia (Spain). BMC Cancer 11: 192.

8. Rue M, Carles M, Vilaprinyo E, Martinez-Alonso M, Espinas JA, et al. (2008) Dissemination of periodic mammography and patterns of use, by birth cohort, in Catalonia (Spain). BMC Cancer 8: 336.
